# Systemic sclerosis in Egyptian population with emphasis on clinical and nailfold capillaroscopic features

**DOI:** 10.1038/s41598-025-14434-w

**Published:** 2025-08-19

**Authors:** Amira Elsonbaty, Manal Hassanien, Abdelhfeez Moshrif, AbdelAzeim Elhefny, Gihan M. Omar, Adel M. Elsayed, Mervat Abo Gabal, Hatem M. Soliman, Rasha A. Abdel-Magied, Sameh Ismail, Mahmoud I. Risha, Hesham Hamoud

**Affiliations:** 1 Rheumatology & Rehabilitation and Physical Medicine Department, Faculty of Medicine, Assiut University, Assuit, Egypt; 2 Rheumatology Department, Faculty of Medicine, Al-Azhar University (Assiut Branch), Assuit, Egypt; 3 Rheumatology Division, Internal Medicine Department, Faculty of Medicine, Ain Shams University, Cairo, Egypt; 4 Rheumatology and Rehabilitation Department, Faculty of Medicine, Minia University, Minya, Egypt; 5 Rheumatology Division, Internal Medicine Department, Faculty of Medicine, Military Medical Academy, Cairo, Egypt; 6 Rheumatology Department, Faculty of Medicine, Al-Azhar University (Cairo branch), Cairo, Egypt

**Keywords:** Diffuse systemic sclerosis, Limited systemic sclerosis, Scleroderma, Capillaroscopy, Egypt, Immunology, Rheumatology

## Abstract

To describe various demographic, clinical, laboratory, and capillaroscopic features of Egyptian patients with systemic sclerosis (SSc) and to explore the relation between various capillaroscopic features and internal organ involvement as well as other disease parameters. In this cross-sectional multi-centric prospective analysis, two hundred twenty-two adult patients with SSc were recruited. Data regarding general and rheumatological examination including the modified Rodnan skin score (MRSS), internal organ involvement and related imaging and laboratory investigations were collected. Both activity and severity indices were measured. Nail fold capillary microscopy (NFC) was performed for 144 patients. Out of 222 patients; 139 (62.6%) had limited type while 83 (37.4%) had diffuse type, middle aged females were predominant (91%). Peripheral vascular, pulmonary, gastrointestinal and general manifestations were the most frequently documented affection with high activity, severity profiles and bad prognostic features in more than half of the patients. MRSS was above 20 in 122 patients (55%), and 126 patients (56.8%) had pulmonary hypertension (SPAP) while 78.4% had ILD which showed a weak correlation to age and disease duration (r = 0.135, r = − 0.152 respectively). Significant weak correlations were found between disease duration and SPAP (r = 0.134), Medseger’s severity score (r = 0.189), peripheral vascular, skin, and GIT affection severity (r = 0.210, 0.135, and 0.196 respectively). Significant difference was observed between different NFC patterns and antibodies, severity scale, some disease manifestations i.e.: general, gastrointestinal, myopathy, and digital ulceration while no significance was found between NFC patterns and the type of SSc. This study provided a comprehensive clinical and laboratory characterization of a large cohort of Egyptian patients with SSc which reflects a more severe disease with unfavorable prognostic aspects compared to the available world-wide reports. Nail fold capillaroscopy showed many associations to different aspects of the disease especially in late and active capillaroscopic features, if introduced early, it may improve the disease outcome.

## Introduction

Systemic sclerosis (SSc) is one of the complex autoimmune diseases that has a distinctive pathology and clinical picture, it’s due to unknown etiology yet the triad of vasculopathy, fibrosis and autoimmune process still the most acceptable profounder of this disease. It usually affects skin but various internal organs could be affected as well including mainly lungs, gastrointestinal tract (GIT), musculoskeletal system, heart, and kidneys^1^.

The prevalence of SSc varies among different populations. In North America, it has been estimated to be (13.5–44.3 per 100,000) and (7.2–33.9 per 100,000 individuals) in Europe. While it’s incidence in both regions is less than 10 per 100,000 individuals with a rapidly progressing manner overtime^2,3^.

There is a gender predilection toward female as most of other autoimmune diseases, where female to male ratio is 3:1 and increases up to 8:1 at late child bearing period, yet the peak incidence occurs around the sixth decade of life^4,5^.

Systemic sclerosis is furtherly distinguished into limited (LSSc) and diffuse (DSSc) forms based on the extent of skin involvement in such patients. LSSc tends to develop Raynaud’s phenomenon (RP), gastroesophageal reflux, pulmonary hypertension (PAH) and occasionally interstitial lung disease (ILD), while the diffuse type causes more systemic affection including arthritis, tendon friction rub, ILD, GIT and myocardial involvement. Moreover, a tendency toward distinguishing antibodies formation occurs in each type, where anticentromere antibodies (ACA) are more likely to be found in LSSc while antitopoisomerase and antipolymerase antibodies are more common in DSSc^3,6^.

Nail fold capillaroscopy (NFC) is a rapid, easy, noninvasive, unexpensive tool that has been long used to assess the peripheral microvasculature changes and to differentiate between patients with primary and secondary Raynaud’s phenomenon with special orientation toward scleroderma; where a unique scleroderma pattern is described by NFC and it includes the presence of hemorrhages, giant capillaries, avascular areas, capillary derangement and neoangiogenic capillaries. Also, staging protocols were proposed in such pattern which includes early, active and late stages according to the percent of each pattern and the derangement of the affected vessels^7–11^.

Some studies documented the association between NFC findings with severity and different internal organs involvement in SSc^12,13^.

Owing to different pathogenetic impact of epidemiological, genetic and environmental factors, ethnicity plays a crucial rule in SSc like most of autoimmune connective tissue diseases. This issue has been previously studied in France and North America, and showed a great variability in different ethnic groups’ profiles^14–16^.

To the best of our knowledge, no wide scale studies have been published on the pattern of SSc in the area of North Africa and Middle East.

In this cross sectional multi-centric prospective analysis, we aimed at documenting different demographic characteristics of the Egyptian systemic sclerosis’ patients together with clinical, laboratory and capillaroscopic features and whether there is a relation between different capillaroscopic features and internal organ involvement and other disease parameters.

## Methods

### Study design and patients’ selection

This cross-sectional study included 222 adult patients with systemic sclerosis diagnosed according to the 2013 American College of Rheumatology/European League Against Rheumatism Systemic Sclerosis classification criteria^17^.

They were recruited from the outpatient clinics and inpatient departments of Rheumatology of the participating University Hospitals during the period from November 2018 to September 2022.

### Ethical considerations

The participating patients gave their written informed consent, and the study protocol was approved by the research ethics committee of Faculty of Medicine, Al-Azhar University, Egypt (REC ID: AZAC/R/85/7-April-2024) and was compatible with the latest version of Helsinki declaration.

### Clinical evaluation and data collection

For all patients, detailed history taking, clinical and rheumatological examinations were performed. The dermal skin thickness was measured by the modified Rodnan skin score (mRSS)^18^. The disease activity was assessed using the European Scleroderma Study Group (EScSG) activity index^19^, and the disease severity was further evaluated using the index of Medsger et al. disease severity scale^20^.

Based on the degree of severity (0: mild-4: very severe) of the following system affection: peripheral vascular system, skin, joints/tendons, muscles, gastrointestinal tract, lung, heart and kidney in addition to the assessment of the patient’s general condition.

Rheumatoid factor (RF), anti-nuclear antibodies (ANA), antitopoisomerase-I antibodies (anti SCL-70) and anticentromere antibodies (ACA) were also estimated. Internal organ affection was assessed according to the patients’ presentation by plain chest X-rays, high resolution CT chest (HRCT) without contrast, electrocardiogram (ECG), and echocardiography.

*Nail fold capillary microscopy*: using (Dino-lite MEDL4N5 pro, video capillaroscope, Netherland) NFC was performed for 144 patients and the presence of the three most common pathological findings were reported as follows: 1-mega capillaries (enlarged loops exceeding normal dimensions; with apical dimension > 50 µm), 2-avascular areas (complete absence of capillary loops over a distance of at least 0.5 mm between two adjacent capillaries) and 3- neo angiogenesis (presence of bushy, tortuous, or ramified capillary formations).

### Statistics

IBM SPSS version 25, Armonk, Y, USA: IBM Corp. was used for statistical analysis. Normal distribution was tested for all quantitative date and accordingly the appropriate test was applied. Normally distributed data were expressed as mean ± standard deviation (SD), while non-normally distributed data were presented as median and interquartile range (IQR). Categorical variables were summarized using frequencies and percentages. Chi square (χ^2^) test was used for comparison of categorical variables or Fisher’s exact test, where appropriate. For continuous variables, group comparisons were carried out using the independent-samples t-test (for normally distributed data) or the Mann–Whitney U test (for non-normally distributed data). Correlations between variables were assessed using Spearman’s rho coefficient for ordinal and non-normally distributed variables. A two-tailed p-value less than 0.05 was considered statistically significant.

## Results

Of 222 Egyptian systemic sclerosis patients that were recruited from departments of Rheumatology of the participating University Hospitals and were treated by government funded healthcare system, 139 patients had limited type and 83 had diffuse SSc. Demographic data are shown in Table 1. Most of the patients were female (91%), middle aged (50.5%), with 1–10 years. disease duration (74.3%). 126 patients (56.8%) had a raised systolic pulmonary arterial pressure (SPAP) with a high probability of pulmonary hypertension according to Jancowish et al.^18^.

**Table 1:** Demographic data and clinical data of studied group.

Items	Patients value n = 222	
Age	Range	18–89 years
	Mean ± SD	42.7 ± 12.4
	Age groups	n (%)
	Young (18– < 40 years)	90 (40.5%)
	Middle (40– < 60 years)	112 (50.5%)
	Old (≥ 60 yrs)	20 (9%)
Gender	Female n (%)	202 (91%)
	Male n (%)	20 (9%)
Disease Duration	Range	1 – 40 yrs
	Mean ± SD	8.3 ± 5.8
	< 5 yrs	64 (28.8%)
	5–10 years	101 (45.5%)
	> 10 years	57 (25.7%)
Type of SSc	LSsc	139 (62.6%)
	DSsc	83 (37.4%)
Autoantibodies	None	19 (8.6%)
	ANA	60 (27%)
	ACA	64 (28.8%)
	Anti-SCL70	19 (8.6%)
	Mixed	60 (27%)
Systemic affection & damage	The most common manifestations:	
	General	161 (72.5%)
	Peripheral vascular	208 (93.7%)
	Joint/Tendon	122 (55%)
	Muscle	120 (54.1%)
	GIT	171 (77%)
	Lung	174 (78.4%)
	Heart	105 (47.3%)
	Renal	30 (13.5%)
MRSS	Range < 20 ≥ 20	0–46 100 (45%) 122 (55%)
EScSG activity index	Active	112 (50.5%)
	Inactive	110 (49.5%)
SPAP on Echo	Within normal (< 30 mmHg)	96 (43.2%)
	Raised(≥ 30 mmHg)	126 (56.8%)
HRCT chest	n = 204	
	ILD changes	130 (63.7%)
	No ILD changes	74 (36.3%)

SD: standard deviation, n (%): number, Frequency, SSc: systemic sclerosis, LSSc: limited scleroderma, DSSc:diffuse scleroderma, ANA:antinuclear antibodies, ACA: anticentromere antibody, Anti SCL-70: antiscleroderma 70 (antitopoisomerase antibody), GIT: gastrointestinal tract, EScSG activity score: the European Scleroderma Study Group activity index, SPAP: systolic pulmonary artery pressure, HRCT: high resolution CT, ILD: interstitial lung diseases.

Lung affection occurred in 78.4% of patients and was documented in 63.7% of patients who performed HRCT chest with varying grades. Demographic and clinical data are shown in Table 1.

We found no significant difference between disease duration and ILD (8.86 ± 5.7, *p* = 0.161), but a significant weak correlation with lung affection severity (r = 190). While ILD-SSc showed a significant difference with different types of SSc. It was 56.2% of LSSc and 43.8% of DSSc.

(*p* = 0.024). Also, there was a significant difference (*p* = 0.000) between ILD-SSc patients with positive antibodies especially ACA (34.6%) and mixed antibodies (ANA &ACA &/or Anti SCL-70 about 30.8%) while ANA alone was positive in 44.6% of patients without ILD.

A weak positive correlation was found between age and both lung and muscle affection severity (r = 0.135, r = − 0.152). Regarding disease duration, significant weak correlations were found to SPAP (r = 0.134), Medseger’s severity score (r = 0.189), peripheral vascular, skin, GIT and.

Lung affection severity (r = 0.210, 0.135, 0.196, 0.190 respectively). Of 144 patients performed NFC, 32 patients had mega capillaries, 33 patients had Avascular area and only 5 patients had Neo-angiogenesis while 73 had mixed patterns of affection especially mega capillaries with avascular area (38.9%). NFC patterns are shown in Table 2.

**Table 2:** Nail fold capilloroscopy patterns.

n = 144		n (%)
Normal		1 (0.7%)
Megacapillaries		32 (22.2%)
Avasculararea		33 (22.9%)
Neoangigenesis		5 (3.5%)
Mixed patterns	Megacaillaries & Avasculararea	56 (38.9%)
	Megacaillaries & Neoangiogenesis	3 (2.1%)
	Avasculararea & Neoangiogenseis	7 (4.9%)
	Megacaillaries & Avasculararea & Neoangiogenesis	7 (4.9%)

n (%): Number, Frequency.

A significant difference was found between capillaroscopy patterns and different antibodies (*p* value = 0.008), and with severity scale of organ involvement (p value = 0.008). We found a significant relation between NFC patterns and general manifestations, muscle and GIT affection (*p* value = 0.042, 0.037, 0.008 respectively), while no significant difference was found with Gender (*p* value = 0.753), Type of SSc (*p* value = 0.877), nor other systems involvement. Data are shown in Table 3 and 4.

**Table 3:** Relation between different quantitative data and NFC patterns.

Items	Megacapillaries Mean ± SD	Avascular area Mean ± SD	Neoangiogenesis Mean ± SD	Mixed patterns Mean ± SD	*P* value
Age	39.1 ± 12.4	47.1 ± 15.9	40.6 ± 11.3	45.6 ± 11.9	0.067
Dis.duration	6.2 ± 4.4	8.3 ± 5.1	6.6 ± 4.04	9.2 ± 7.2	0.160
MRSS	22.6 ± 11.98	23.6 ± 9.7	16.0 ± 12.9	20.9 ± 11.2	0.207
SPAP	31.98 ± 9.6	32.7 ± 9.7	33.0 ± 5.2	31.2 ± 10.6	0.816
EScSG activity index	2.6 ± 1.4	3.3 ± 2.0	2.7 ± 2.1	2.9 ± 1.7	0.605
Medseger’s severity score	9.1 ± 3.4	8.03 ± 3.1	10.4 ± 4.03	9.34 ± 3.1	**0.008**

SD: standard deviation, Dis.:disease, MRSS: modified Rodnan’s skin score, SPAP: systolic pulmonary artery pressure, EScSG activity score: the European Scleroderma Study Group activity index score. One way ANOVA test was applied. Significant values are in [bold].

**Table 4:** Relation between different categorical data and NFC patterns.

Item		Megacapillaries n (%)	Avascular area n (%)	Neoangiogenesis n (%)	Mixed patterns n (%)	*P* value
Antibodies	ANA	14 (31.1%)	5 (11.1%)	0 (0%)	26 (57.8%)	**0.008**
	ACA	9 (24.3%)	15 (40.5%)	2 (5.4%)	11 (29.7%)	
	AntiScl-70	2 (22.2%)	5 (55.6%)	0 (0%)	2 (22.2%)	
	Mixed antibodies	5 (11.6%)	5 (11.6%)	2 (4.7%)	30 (69.8%)	
Different affected systems	General	23 (23.5%)	4 (4.1%)	16 (16.3%)	55 (56.1%)	**0.042**
	Peripheral vascular	31 (22.8%)	4 (2.9%)	30 (22.1%)	71 (52.5%)	0.200*
	Joint/tendon	18 (26.5%)	1 (1.5%)	20 (29.4%)	29 (42.6%)	0.088
	Muscle	16 (24.2%)	3 (4.5%)	8 (12.1%)	39 (59.1%)	**0.037**
	GIT	23 (22.5%)	4 (3.9%)	16 (15.7%)	59 (57.8%)	**0.008**
	Lung	22 (20%)	4 (3.6%)	26 (23.6%)	58 (52.7%)	0.669
	Heart	10 (20.4%)	3 (6.1%)	12 (24.5%)	24 (49%)	0.631
	Kidney	3 (17.6%)	1 (5.9%)	1 (5.9%)	12 (70.6%)	0.219

Correlation between disease duration and different systems’ affection severity**			Correlation between age and different systems’ affection severity**		
Peripheral vascular	r = 0.210	*P* = 0.002	lung affection	r = 0.135	*P* = 0.044
Skin	r = 0.135	*P* = 0.045	Muscle affection	r = − 0.152	*P* = 0.023
GIT	r = 0.196	*P* = 0.003			
Lung affection	r = 0.190	*P* = 0.005			
SPAP	r = 0.134	*P* = 0.046			
Medseger’s severity score	r = 0.189	*P* = 0.005			

n (%): number, Frequency. Chisquare test was applied, *only digital ulcer was significant with different capillaroscopic features, **Spearman’s correlate. Significant values are in [bold].

Lastly, a significant difference (*p* value = 0.000) was found between digital ulceration and different NFC patterns especially mixed pattern, where microangiopathy with avascular area were manifested in 40/75 patients with digital ulcers.

## Discussion

Ethnicity plays a crucial role in the pathogenesis, clinical presentation and outcome of SSc as reported by many studies^14–16^. The PHAROS registry has concluded the presence of more severe SSc related ILD in Caucasian population (64%) compared to black /African -Americans (24%) and Hespanics (11%)^21^.

Recently, 190 patients of various ethnic groups were included in UAE registry, 90 patients were purely of emirates origin. Prevalence of SSc was estimated to be 1.66/100,000 for total registry and 7.78/100,000 for emirates origin patents. Females were predominant. DSSc was slightly more predominant (54.5%), mean age of emirates origin patients at time of diagnosis was 34 ± 11 for DSSc and 41 ± 14 for LSSc. More than 92% of patients were ANA positive, 48.6% were Anticentromere positive more at LSSc, and 64.4% had positive AntiSCL70 more at DSSc. Prevalence of patients with both ILD and PAH was 1.12/100,000 among emirates origin patients^22^.

Our study was on exclusively Egyptian SSc patients, female patients were also predominant (91%), aged from 18 to 89 years, where the largest sector to be in their middle age (50.5%) similar to the previous registry. LSSc was more evident than diffuse type and the disease duration reached 40 years in one patient but most of patients were having a duration between 5 and 10 years (45.5%). 91.4% of patients had positive antibodies especially ACA and ANA. The most frequent systems involved were peripheral vascular, pulmonary, gastrointestinal, and musculoskeletal systems with marked general manifestations noticed among our patients. Unlike international incidence and prevalence of cardiac and pulmonary hypertension in SSc patients as stated to reach 12%^23^, about half of our patients had cardiac affection with high probability of PAH. The modified Rodnan’s skin score was above 20 in 55% of patients. These observations indicate high disease severity and poor prognosis, potentially influenced by limited access to specialized care and socioeconomic disadvantage, which may affect treatment compliance in some regions of Egypt.

Focusing on the interstitial lung disease that complicates 10–15% of SSc patients in usual^24^, our patients’ were affected by ILD with varying grades revealed by HRCT chest reaching up to 63.7%. Previous studies had reported a significant relation between ILD- SSc and longer disease duration^25–27^.

Contrarily, we found no correlation between duration and ILD. However, we found a significant difference when it came to the type of SSc. LSSc was more complicated with ILD as previously reported by many studies^25,27,28^.

Notably, there was a significant difference between ILD- SSc and positive serology as patients with ACA showed lesser ILD, and those with Mixed Antibodies (ANA &ACA &/or Anti SCL-70) were highly affected.

Nail fold capillaroscopy showed a great help in the diagnosis of the early stages of vascular affection according to many literatures and can be used as a screening tool for early diagnosis of scleroderma patients especially those not completing the criteria but primarily presenting with Raynaud’s phenomenon. Moreover, it has been included in the 2013 ACR/EULAR classification criteria and VEDOS classification criteria.^17,29^**.**

Also, NFC patterns can predict disease activity and strongly correlate to many internal organs’ affection as ILD, PHT, GIT, musculoskeletal, and renal involvement^30–36^.

Although capillaroscopic patterns were not quantitatively analyzed, we retrospectively referenced Cutolo et al.’s classification, categorizing patients as early (minimal changes), active (frequent megacapillaries and moderate avascularity), or late (extensive avascular areas and neoangiogenesis).

**Fig. 1:**
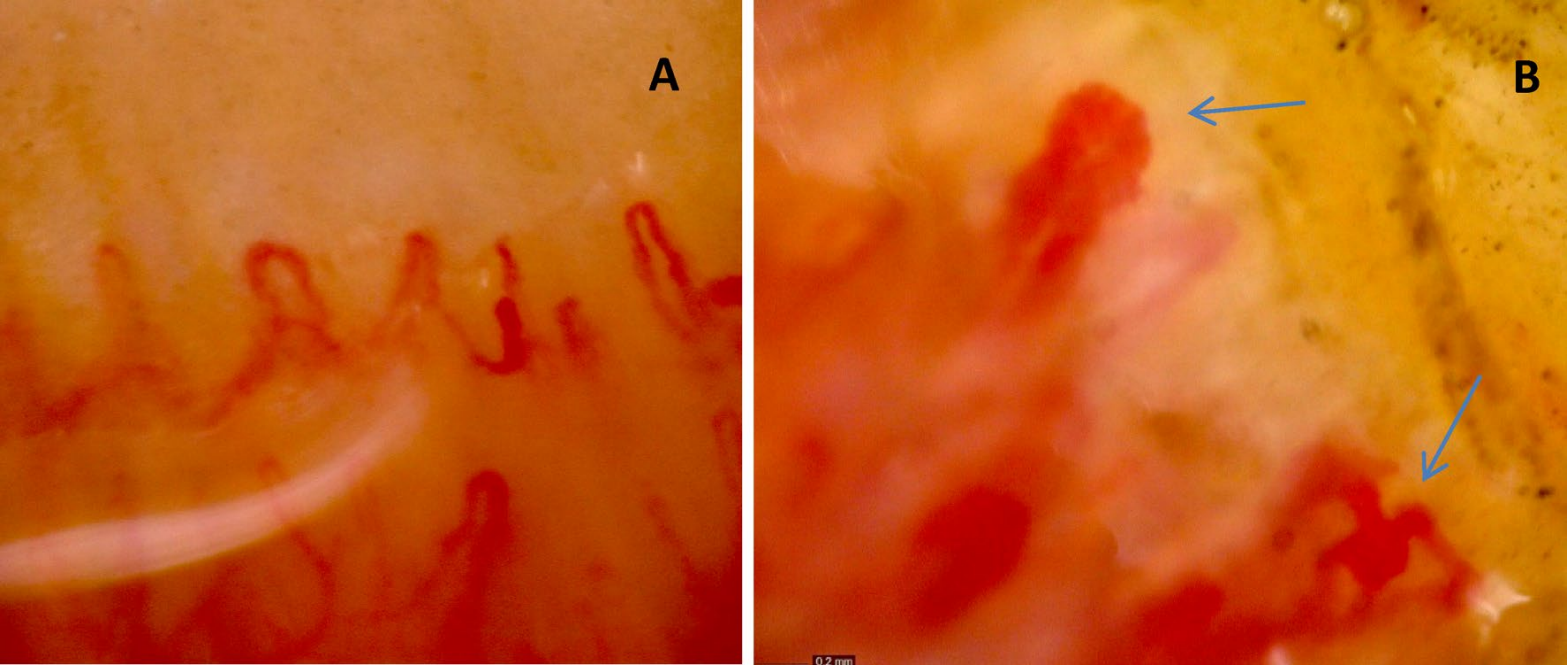
Nailfold capillaroscopic image from a patient with systemic sclerosis showing: (**A**) avascular area (gap > 0.5 mm), (**B**) prominent megacapillaries ( apical dimension ≥ 50 µm), and neoangiogenic capillary loops (bushy, tortous capillary formation). Image acquired at × 200 magnification. Scale bar: 0.2 mm. These finding highlights significant microvascular alterations associated with systemic sclerosis.

Our patients exhibited frequent NFC abnormalities, mainly megacapillaries and avascular areas (Fig. 1A and B), indicating either active or late stages of grading among most of examined patients and rarity of early stages.

The majority of patients exhibited prominent NFC abnormalities, primarily megacapillaries and avascular areas (Fig. 1A and B), reflecting features of either active or late scleroderma patterns. Although a quantitative analysis of capillaroscopic patterns was not performed, we retrospectively applied the classification by Cutolo et al.^8^, where the early pattern reflects minimal alterations, the active pattern is defined by frequent megacapillaries and moderate avascularity, and the late pattern denotes extensive avascular zones and neoangiogenesis. Early-stage findings were rarely observed within our cohort.

We found a significant difference between NFC patterns and positive antibodies (especially Mixed antibodies). ANA was more prevalent in mega capillaries while ACA was more in avascular area finding. Moreover, patients with mixed capillaroscopic features usually had more than one antibody (69.8%). This is in agreement with Ingegnoli et al. who stated a strong relation between different antibodies and capillaroscopic patterns especially in late stages^34^.

Cutolo et al. and Caramaschi et al., found different stages of NFC to be more profound in limited type SSc especially those with early and active pattern, while late stages are more common in diffuse type^33,37^.

In contrary, we found no significance between both SSc subtypes and different capillaroscopic features and that was in agreement with Shenavandeh, et al. in their study on 70 patients with SSc^35^.

Caramaschi et al., reported a correlation of microangiopathy with disease severity and different systematic affection including lung, heart, skin and peripheral vasculopathy^33^. While Kenik et al. didn’t find a significant relation between stages of skin affection and capillary changes by NFC^38^.

Bredemeier et al. documented association between avascular area finding in NFC and disease duration, skin score, GIT, peripheral ischemia and ILD^30^.

In our study, a significant difference was shown between capillaroscopic features and disease’ severity, some visceral affection including muscle, gastrointestinal affection, and digital ulceration, while no significance was found with age, disease duration, activity, Modified Rodnan’s skin score nor other affected systems.

Digital ulceration was found mainly in mixed patterns affection of NFC, especially microangiopathy with avascular area that was manifested in 40/75 patients with digital ulcers. The importance of this technique which was studied thoroughly by 5 prospective studies in a trial to know the exact relation between different capillaroscopic changes and occurrence and healing of digital ulcers^36,39–43^.

Other studies reported the presence of certain types of NFC patterns and more late stages in patients with PAH in comparison to those without. Also, they found increased score of avascular area (> 1–2) in PAH patients^27,44^.

In contrary, Greidinger et al. didn’t find any association between PAH and different capillaroscopic patterns^45^.

Similarly other studies reported a relation between ILD-SSc and active or late NFC patterns, especially with high score of avascular area^27,46^.

Moreover, others reported the presence of decreased capillary density and increased capillary width in patients with ILD^27,47–49^.

In our study, we didn’t find a significant difference between either ILD or increased SPAP and different capillaroscopic features. This may be explained by different statistical methods used in different studies and different sample sizes.

Further longitudinal prospective and multiethnic studies are needed to disentangle the relative contributions of genetic background, environmental exposures, and healthcare disparities in shaping SSc outcomes. Also, using the standards of nail fold capillaroscopy recommended by the latest Delphi consensus and/or Cutolo et al. staging could enhance data reproducibility and support future clinical comparisons.

## Conclusion

This study provided a comprehensive clinical and laboratory characterization of a large cohort of Egyptian patients with SSc which reflects a more severe disease with unfavorable prognostic aspects compared to the available world-wide reports. Nail fold capillaroscopy showed many associations to different aspects of the disease especially in late and active capillaroscopic patterns. To our knowledge, this is the first study to comprehensively correlate these capillaroscopic features with clinical manifestations in an Egyptian SSc cohort, underscoring its novelty. These results suggest that the early integration of nailfold capillaroscopy into routine assessment may enhance risk stratification and guide timely therapeutic interventions**,** and hence the disease outcome especially in resource-limited settings.

## Data Availability

No datasets were generated or analysed during the current study.

## Abbreviations

ACA: Anticentromere antibodies
ANA: Anti-nuclear antibodies
Anti SCL-70: Antitopoisomerase I antibodies
CBC: Complete blood count
CRP: C-reactive protein
DSSc: Diffuse systemic sclerosis
ECG: Electrocardiogram
EScSG: European Scleroderma Study Group Activity Index
ESR: Erythrocyte sedimentation rate
GIT: Gastrointestinal tract
HRCT: High resolution CT chest
ILD: Interstitial lung disease
LSSc: Limited systemic sclerosis
MRSS: Modified Rodnan skin score
NFC: Nail fold capilloroscopy
PAH: Pulmonary hypertension
PFTs: Pulmonary function tests
RF: Rheumatoid factor
RP: Raynaud’s phenomenon
SSc: Systemic sclerosis
